# Conservative versus liberal oxygen therapy in hypoxic ischemic encephalopathy following cardiac arrest: a trial-based cost-effectiveness analysis

**DOI:** 10.1016/j.resplu.2026.101403

**Published:** 2026-07-09

**Authors:** Zhomart Orman, Carol Hodgson, Michael Bailey, Diane Mackle, Anne M Mather, Paul Young, Alisa M. Higgins, LOGICAL Investigators and the Australian and New Zealand Intensive Care Society Clinical Trial Group

**Affiliations:** a Australian and New Zealand Intensive Care Research Centre, School of Public Health and Preventive Medicine, Monash University, 509 St Kilda Road, Melbourne, Victoria 3004, Australia; b Medical Research Institute of New Zealand, Level 7, CSB Building, Wellington Hospital, Riddiford St, Newtown, Wellington 6021, New Zealand; c Intensive Care Medicine, Wellington Hospital, Riddiford Street, Newtown, Wellington 6021, New Zealand

**Keywords:** Cost-effectiveness, Economic evaluation, Cardiac arrest, Oxygenation therapy, Intensive care, Hypoxic ischemic encephalopathy

## Abstract

**Background:**

The cost-effectiveness of conservative versus liberal oxygen therapy in critically ill adults with post-cardiac arrest hypoxic ischemic encephalopathy is unknown.

**Methods:**

A cost-effectiveness analysis alongside the LOGICAL trial was conducted from a health system perspective. Conservative oxygen targeted arterial oxygen saturation (SpO_2_) of 90–95% using the lowest fraction of inspired oxygen (FiO_2_), while liberal oxygen had no upper SpO_2_ limit and a minimum FiO_2_ of 0.3 during invasive ventilation. Patient-level data were analyzed from 53 intensive care units (ICU) in Australia, New Zealand, and Ireland. Costs (2024, US$) included ICU, ward, and inpatient rehabilitation stays. Health outcomes included favorable functional outcome (Glasgow Outcome Scale-Extended score 5–8) and quality-adjusted life years (QALYs) at 180 days derived from EQ-5D-5L. Uncertainty was explored using bootstrapping. Discounting was not applied.

**Results:**

Among 1821 participants (median age 62 years; 28.6% female), 873 were randomized to conservative and 948 to liberal oxygen. Mean per-patient cost was $33,629 ($33,517) in the conservative group, with an incremental cost of −$1392 (95% CI, −$4696 to $1911). Favorable functional outcome occurred in 38.2% with conservative oxygen (risk difference, −1.5 percentage points; 95% CI, −6.1 to 3.2). Incremental QALYs were −0.002 (95% CI, −0.013 to 0.008). Conservative oxygen was associated with cost-savings of $635,151 per QALY forgone with considerable uncertainty (>55% bootstrapped replications). Incremental net monetary benefit was $1173 (95% CI, −$2096 to $4442) at $100,000 per QALY.

**Conclusions:**

Conservative oxygen was associated with modest cost savings and small differences in health outcomes, with no clear economic advantage over liberal oxygen.

**Trial number:** ACTRN12621000518864.

## Highlights

• Healthcare cost of post-cardiac arrest hypoxic ischemic encephalopathy is considerable.
• Cost-effectiveness of conservative versus liberal oxygen therapy in cardiac arrest is unclear.
• Conservative oxygen is associated with modest cost savings and small differences in health outcomes.
• Conservative oxygen has no clear economic advantage over liberal oxygen.

## Introduction

Cardiac arrest is a global public health concern. Out-of-hospital cardiac arrest (OHCA) affects an estimated 3.8 million people annually, with only 10% surviving to hospital discharge.^1^, ^2^ Among those who survive to hospital discharge or 30 days after OHCA, 77% remain alive at one year, of whom 83% achieve favorable neurological outcomes.^3^ Consequently, cardiac arrest contributes significantly to mortality and health loss worldwide, necessitating effective, evidence-based treatments.

The economic burden of cardiac arrest is considerable. In the United States (US), the mean cost of index hospitalization among 26,900 patients with OHCA was US$23,300, including those who died, and US$32,400 among those who survived to discharge between 2013 and 2019.^4^ In Australia, annual national losses attributable to cardiac arrest are estimated to approach US$1.42 billion, comparable to productivity losses from all cancers combined.^5^ In Europe, healthcare costs per hospital survivor have been reported to range from €50,000 to €81,000.^6^, ^7^, ^8^, ^9^ These costs, combined with the high mortality and morbidity, underscore the need for cost-effective interventions.

Supplementary oxygen, delivered via invasive or non-invasive ventilation, is used to prevent and treat hypoxic ischemic encephalopathy (HIE), a brain injury resulting from reduced blood flow after cardiac arrest. However, whether higher levels of fraction of inspired oxygen (FiO_2_) and arterial oxygen saturation measured by pulse oximetry (SpO_2_) improve survival or functional recovery remains unclear. A meta-analysis of seven randomized controlled trials (429 patients) with post-cardiac arrest HIE reported a trend toward improved survival up to one year with conservative versus liberal oxygen therapy.^10^ More recently, the LOGICAL trial of 1821 patients with HIE after cardiac arrest found no difference in survival with favorable functional outcome between conservative and liberal oxygen strategies.^11^ To date, the cost-effectiveness of these oxygen strategies has not been reported. We therefore assessed the cost-effectiveness of conservative versus liberal oxygen therapy to inform clinical and policy decision-making.

## Methods

This was a trial-based cost-effectiveness analysis comparing conservative with liberal oxygen therapy, as prespecified in the LOGICAL trial protocol^12^ and health economic analysis plan.^13^ Reporting followed the Consolidated Health Economic Evaluation Reporting Standards (CHEERS) guidelines.^14^ The economic evaluation was conducted according to intention-to-treat principles from a health system perspective, specifically that of a government funder focusing on inpatient healthcare services. Because follow-up was limited to 180 days, costs and benefits were not discounted.

### Trial design and participants

LOGICAL was a multicenter, blinded, randomized controlled trial conducted in 53 intensive care units (ICUs) across Australia, New Zealand, and Ireland.^11^, ^12^ The trial compared conservative with liberal oxygen therapy in critically ill adults (≥18 years) with suspected post-cardiac arrest HIE who were receiving invasive mechanical ventilation in the ICU. Patients were excluded if enrolment was not considered to be in the patient’s best interest by the treating clinician, if they had previously been enrolled in the Mega-ROX trial (in which the LOGICAL trial was nested), or if more than 12 h had elapsed since fulfillment of the inclusion criteria.^15^, ^16^ The primary outcome was a favorable functional outcome at 180 days post-randomization. For participants alive at follow-up, assessments were completed when feasible rather than at a fixed calendar date, consistent with pragmatic trial conduct.

### Interventions

The intervention was conservative oxygen therapy, with FiO_2_ reduced as rapidly as possible to a minimum of 0.21 (room air) while maintaining SpO_2_ above the acceptable lower limit (default 91%, adjustable at clinician discretion). SpO_2_ levels above 94% were actively avoided, with an upper SpO_2_ alarm limit of 95% to minimize the risk of hyperoxemia. The comparator was liberal oxygen therapy, in which no specific measures were taken to avoid high FiO_2_ or SpO_2_ levels. The use of upper SpO_2_ alarm limits was prohibited, and the minimum acceptable FiO_2_ was set at 0.30 for invasively ventilated patients.

### Health outcomes

Health outcomes included favorable functional outcome, quality-adjusted life years (QALYs), and life years, all assessed at 180 days post-randomization. Functional outcome was measured using the Glasgow Outcome Scale Extended (GOS-E),^17^ which categorizes patient outcomes into eight levels: dead, vegetative state, lower and upper severe disability, lower and upper moderate disability, and lower and upper good recovery. A favorable functional outcome was defined as a GOS-E score of 5–8, indicating recovery to at least lower moderate disability, while unfavorable functional outcome was defined as a GOS-E score of 1–4.

QALYs were estimated from EQ-5D-5L-derived utilities over 180 days using Australian value sets, with 0 representing death and 1 representing full health.^18^, ^19^, ^20^ The area under the curve (AUC) approach was used to calculate QALYs, assuming zero utility at randomization and a linear interpolation between randomization and 180-day EQ-5D-5L assessment.^21^, ^22^ Because EQ-5D-5L was assessed only at 180 days and not before death, utility trajectories prior to death were unavailable. Consistent with the pre-specified health economic analysis plan, participants who died before 180 days were assigned a utility value of zero at 180 days. Consequently, QALY estimates reflected survival status and EQ-5D-5L-assessed health-related quality of life at 180 days rather than the timing of death. Life-years were estimated separately using restricted mean survival time truncated at 180 days, with survival time converted from days to years.^23^

### Cost estimation

Costs were estimated by assigning unit costs (Supplementary Table 1) to patient-level resource use from case report forms (CRFs) and the Adult Patient Database (APD) of the Australian and New Zealand Intensive Care Society.^24^ The CRFs captured the index hospitalization details, including ICU admission and discharge dates along with baseline participant characteristics (e.g., age, sex), clinical information related to cardiac arrest (cause, date, and time), and details of oxygen therapy. The APD provided discharge destination data for individual ICU episodes in Australia and New Zealand. Comparable data from Ireland was obtained via REDCap. Admission and discharge dates were cross-checked across sources for consistency.

Healthcare resource use from randomization up to 180 days or death, whichever occurred first, was based on the duration of the index ICU stay, subsequent hospital ward stay, and inpatient rehabilitation (*Length of hospital stay measurement* in Supplementary Appendix). Inpatient costs account for 93% of total costs of patients with OHCA from a cardiac arrest up to 30 day or death.^25^ Costs of oxygen were not included in the analysis because medical oxygen represents a negligible proportion of total inpatient costs in the intensive care setting, and any between-group differences in oxygen consumption were unlikely to have a meaningful impact on overall cost estimates.^26^ All costs were reported in US dollars ($), converted from Australian dollars, New Zealand dollars, and euros for Ireland using 2024 purchasing power parity (PPP) conversion factors published by the World Bank.^27^ Where necessary, costs were adjusted for inflation using country-specific health price indices.^28^, ^29^, ^30^

### Cost-effectiveness outcomes

The cost-effectiveness of conservative versus liberal oxygen therapy was assessed using several complementary approaches. First, mean total per-patient costs and health outcomes were compared between treatment groups. Second, incremental cost-effectiveness ratios (ICERs) were calculated as the difference in mean total per-patient costs (incremental cost) divided by the corresponding difference in health outcomes (incremental effect). Cost-effectiveness outcomes included cost per favorable functional outcome, cost per QALY, and cost per life-year gained.

Additionally, incremental net monetary benefit (INMB) was calculated at willingness-to-pay thresholds of $50,000, $100,000, and $200,000 per QALY, defined as the monetary value of incremental health gains minus incremental costs.^31^ Uncertainty surrounding cost-effectiveness estimates was explored using non-parametric bootstrapping, with results presented on cost-effectiveness planes. The probability of cost-effectiveness across a range of willingness-to-pay thresholds was illustrated using a cost-effectiveness acceptability curve.

### Statistical analysis

All analyses were conducted using Stata Statistical Software (StataCorp, 2025: Release 19.5, College Station, TX). Baseline characteristics were summarized as proportions for all categorical variables, and median with interquartile ranges (IQR) for continuous variables. Healthcare resource utilization, costs, and health outcomes were presented by treatment group as means with standard deviations (±) or as counts with percentages (%), consistent with cost-effectiveness outcome calculations. Between-group differences were presented with 95% confidence intervals (95% CI) using linear regression with robust standard errors for continuous outcomes and generalized linear models with a binomial family and identity link for binary outcomes. Missing data were characterized descriptively and were handled using multiple imputation by chained equations (*Handling missing data* in Supplementary Appendix).

Cost-effectiveness outcomes were analyzed using complete case data. To assess uncertainty in cost-effectiveness estimates, probabilistic sensitivity analysis using non-parametric bootstrapping with 10,000 replications was performed to jointly estimate incremental costs and incremental effects with 95% CI. The resulting bootstrap replications were plotted on a cost-effectiveness plane, a scatter plot divided into four quadrants defined by the axes of incremental cost (vertical axis) and incremental effectiveness (horizontal axis). The north-east quadrant represents interventions that are more costly and more effective; the south-east quadrant represents those that are less costly and more effective (dominant); the south-west quadrant represents those that are less costly and less effective; and the north-west quadrant represents interventions that are more costly and less effective (dominated). Proportions of bootstrapped replications in each quadrant were also presented.

Sensitivity analyses using alternative rehabilitation ascertainment based on grouped discharge-destination categories (*Inpatient rehabilitation measurement* in Supplementary Appendix), along with prespecified subgroup analyses, were conducted to explore heterogeneity in cost-effectiveness estimates by cardiac arrest cause (medical versus non-medical); arrest location (out-of-hospital versus in-hospital); and initial rhythm (shockable versus non-shockable).

### Ethics

Ethics approval was obtained from all participating jurisdictions or institutions (Australia: 2020/ETH00961, 11/08/2021; New Zealand: 19/NTB/195, 24/03/2021; Ireland: 21-002-AFI, 8/082022). Because all patients enrolled in the trial lacked decision-making capacity at the time of enrolment, several consent pathways were permitted: (i) a priori consent from a substitute decision maker; (ii) delayed consent from a substitute decision maker; (iii) delayed consent for continued participation from the patient; (iv) waiver of consent; (v) consent provided by an ethics committee, guardianship board, or other legal authority; and (vi) opt-out consent. The availability of specific consent options at individual participating sites was determined by the relevant ethics committees and/or administrative tribunals and was subject to applicable laws in each participating country. Further details regarding trial methods, secondary outcomes, and data collection are available in the published trial protocol.^12^

## Results

### Baseline characteristics

Among the 1821 trial participants (median age, 62 years [IQR 52–72]; 28.6% female), 873 (47.9%) were randomized to the conservative oxygen group and 948 (52.1%) to the liberal oxygen group. Overall, baseline characteristics were well balanced between the groups (Table 1).

**Table 1:** Baseline characteristics of study participants.

**Characteristic**	**Conservative oxygen** **(*n* = 873)**	**Liberal oxygen** **(*n* = 948)**
Age, median (interquartile range) years	61 (52–71)	63 (53–72)
Female, *n* (%)	244 (27.9)	277 (29.2)
**Country, *n* (%):**		
Australia	632 (72.4)	650 (68.6)
New Zealand	238 (27.3)	298 (31.4)
Ireland	3 (0.3)	0
**Arrest cause, *n* (%):**		
Medical	772 (88.4)	823 (86.8)
Non-medical	101 (11.6)	125 (13.2)
**Most prevalent diagnosis, *n* (%):**		
Ventricular tachycardia or fibrillation	232 (26.6)	277 (29.2)
Acute myocardial infarction	156 (17.9)	154 (16.2)
Pulseless electrical activity	90 (10.3)	119 (12.6)
Other	395 (45.2)	398 (42.0)
**Cardiac arrest location, *n* (%):**		
Out-of-hospital	647/868 (74.5)	698/941 (74.2)
In-hospital	221/868 (25.5)	243/941 (25.8)
**First monitored rhythm, *n* (%):**		
Shockable	423/829 (51.0)	477/903 (52.8)
Not shockable	406/829 (49.0)	426/903 (47.2)

At 180 days, GOS-E data were available for 1709 participants (94%), EQ-5D-5L data for 1692 participants (93%), and survival data for 1809 (99%) (Supplementary Figs. 1–3). As missingness for favorable functional outcome was reported in the main trial publication, EQ-5D-5L missingness is reported in Supplementary Tables 2, 3. Missing life year data at 180 days were <1% and were therefore not reported separately.

### Health outcome summaries

A favorable functional outcome was observed in 38.2% of participants in the conservative oxygen group and 39.7% in the liberal oxygen group (risk difference, −1.5 percentage points; 95% CI, −6.1 to 3.2). Mean QALYs were 0.092 (0.109) in the conservative oxygen and 0.094 (0.110) in the liberal oxygen, with a between-group difference of −0.002 (95% CI, −0.013 to 0.008). The incremental mean survival time was −2.9 days (−0.008 life-years; 95% CI, −0.030 to 0.014).

### Healthcare resource utilization and costs

The point estimates for ICU and hospital ward lengths of stay were similar between groups, with a mean difference of −0.2 days (95% CI, −0.7 to 0.3) for ICU stays and −0.1 days (95% CI, −1.3 to 1.1) for medical ward stays. Similarly, the between-group difference in the proportions of participants who received inpatient rehabilitation was not statistically significant (Table 2).

**Table 2:** Healthcare resource utilization, costs, and health outcomes at 180 days.

**Outcome**	**Conservative oxygen** **(*n* = 873)**	**Liberal oxygen** **(*n* = 948)**	Between-group difference (95% CI)*
**Healthcare resource utilization**			
Length of ICU stay, mean (SD)	5.0 (5.1)	5.2 (5.7)	−0.2 (−0.7 to 0.3)
Length of medical ward stay, mean (SD)	6.8 (12.1)	6.9 (13.8)	−0.1 (−1.3 to 1.1)
Inpatient rehabilitation, *n* (%)	69 (7.9)	63 (6.7)	1.3 (−1.1 to 3.7)
**Per-patient costs, mean (SD)**			
ICU	$23,162 ($24,307)	$24,529 ($26,762)	−$1367 (−$3703 to $968)
Medical ward	$9604 ($17,018)	$9805 ($19,820)	−$201 (−$1895 to $1493)
Inpatient rehabilitation	$1175 ($4018)	$999 ($3752)	−$176 (−$182 to $534)
Total	$33,629 ($33,517)	$35,263 ($40,784)	−$1392 (−$4696 to $1911)
**Health outcomes**			
Favorable functional outcome (GOS-E 5–8), *n* (%)	313/819 (38.2)	353/890 (39.7)	−1.5% (−6.1% to 3.2%)
QALYs, mean (SD)†	0.092 (0.109)	0.094 (0.110)	−0.002 (−0.013 to 0.008)
Survival time, mean (SE), days‡	90.9 (3.0)	93.8 (2.8)	−2.9 (−10.9 to 5.2)

*Abbreviations:* CI, confidence interval; GOS-E, Glasgow Outcome Scale Extended; ICU, intensive care unit; SD, standard deviation; SE, standard error; QALYs, quality-adjusted life years.

* The difference in percentages was calculated using generalized linear model with a binomial family and an identity link; and the difference in means were calculated using linear regression with robust standard errors.

† QALYs were calculated by linear interpolation from baseline (utility = 0) to 180 days using EQ-5D-5L utility data for 813 participants in the conservative oxygen and 879 participants in the liberal oxygen. Participants who died before 180 days were assigned a QALY of 0.

‡ Survival time was calculated as the restricted mean survival time, truncated at 180 days.

The mean total per-patient cost was $33,629 ($33,517) in the conservative oxygen group and $35,263 ($40,784) in the liberal oxygen group, representing an incremental cost of −$1392 (95% CI, −$4696 to $1911) (Table 2). ICU stay costs accounted for 68% of total mean per-patient costs, medical ward stays for 28%, and inpatient rehabilitation for 4%.

### Cost-effectiveness summaries

**Fig. 1:**
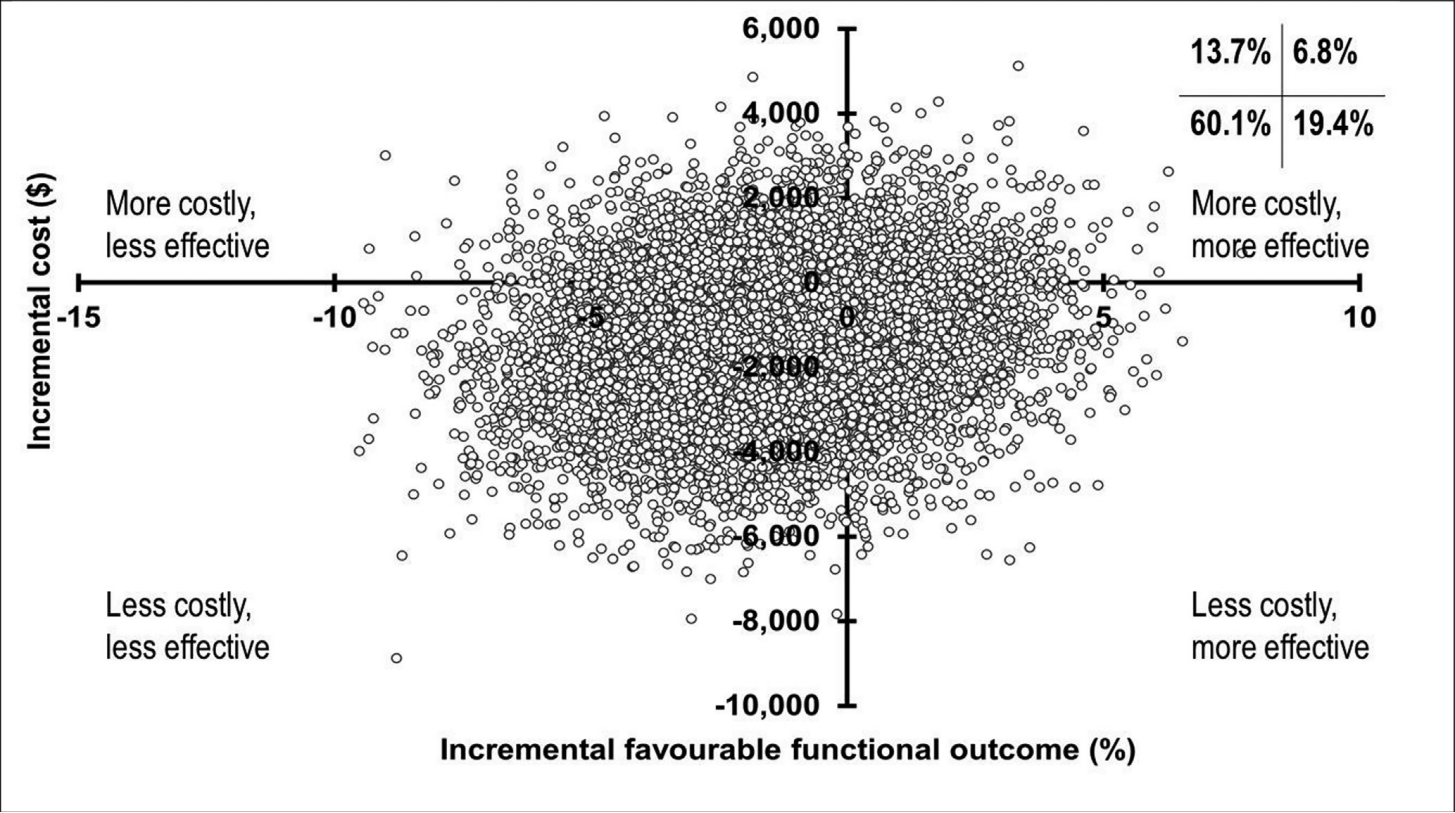
**Cost-effectiveness plane of incremental costs and incremental favorable functional outcome**.

**Fig. 2:**
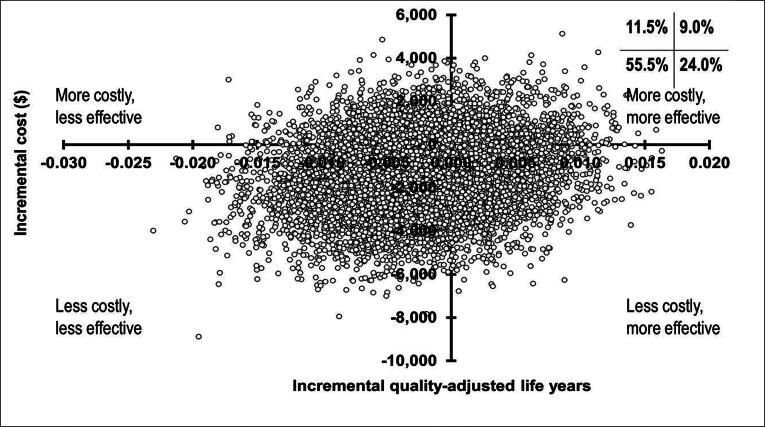
**Cost-effectiveness plane of incremental costs and incremental quality-adjusted life years**.

**Fig. 3:**
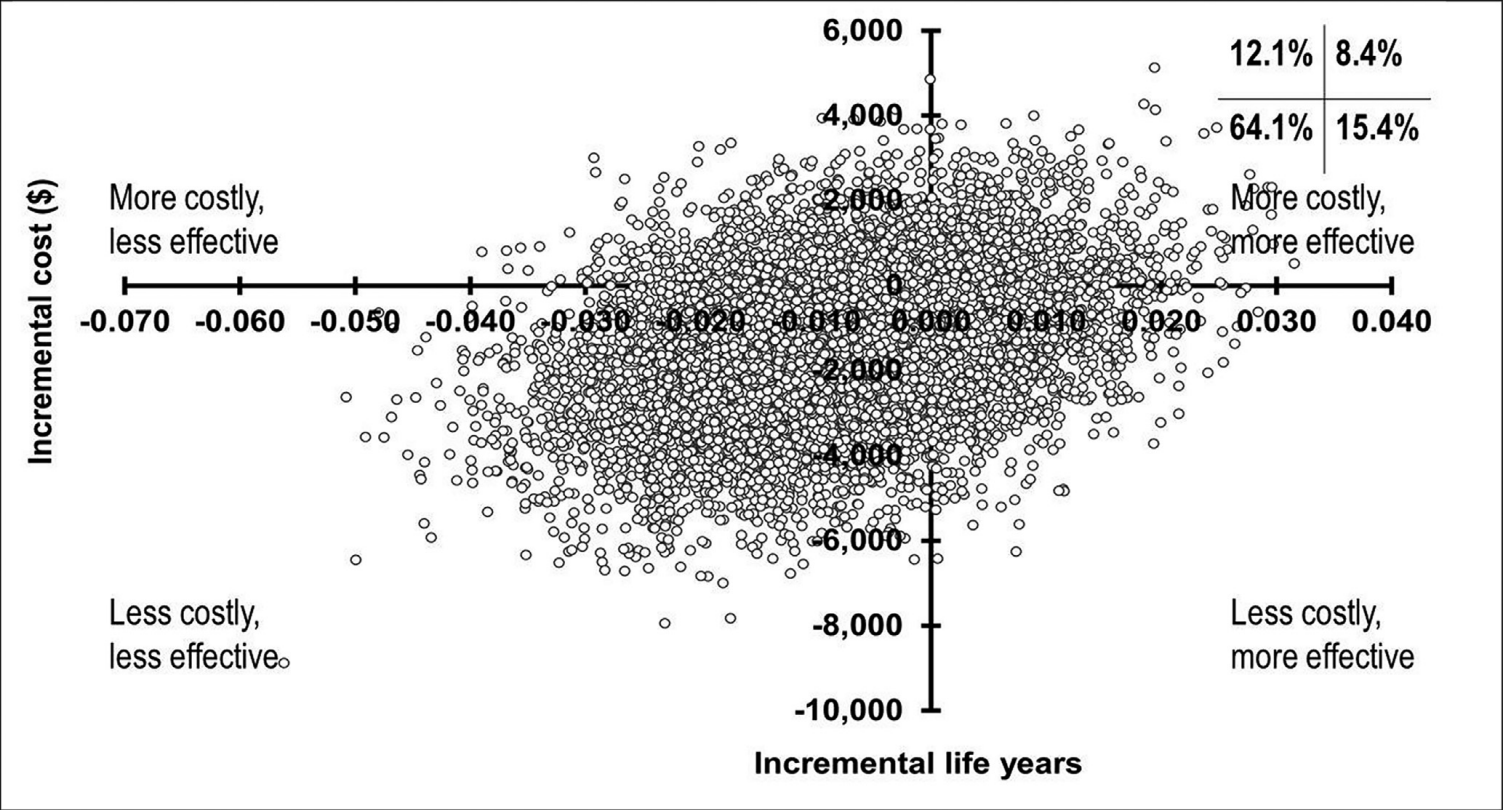
**Cost-effectiveness plane of incremental costs and incremental life years**.

The point estimates of the ICERs indicated that conservative oxygen was less costly but also marginally less effective than liberal oxygen. Estimated cost-savings were $96,306 per favorable functional outcome forgone, $635,151 per QALY foregone, and $177,973 per life-year forgone (Table 3). Most bootstrapped replications fell in the south-west quadrant of the cost-effectiveness planes (Fig. 1, Fig. 2, Fig. 3), accounting for 60.1% of replications for favorable functional outcome, 55.5% for QALYs, and 64.1% for life-years. This pattern reflects modest cost savings accompanied by small reductions in health outcomes. Importantly, the majority of these replications were concentrated close to the origin, with incremental costs generally within −$5000 and incremental QALYs within −0.015, indicating the differences in both costs and QALYs were small in magnitude.

**Table 3:** Summary of cost-effectiveness outcomes.

**Cost-effectiveness outcome**	**Incremental cost (95% CI)**	**Incremental effect (95% CI)**	**ICER ($ per unit lost)**
Cost per favorable functional outcome	−$1392 (−$4696 to $1911)	−1.5% (−6.1% to 3.2%)	$96,306 saved per favorable functional outcome forgone
Cost per QALY	−$1392 (−$4696 to $1911)	−0.002 (−0.013 to 0.008)	$635,151 saved per QALY forgone
Cost per life-year	−$1392 (−$4696 to $1911)	−0.008 (−0.030 to 0.014)	$177,973 saved per life-year forgone

*Abbreviations:* ICER, incremental cost-effectiveness ratio; INMB, incremental net monetary benefit; QALY, quality-adjusted life-year.

The INMB was $1283 (95% CI, −$1961 to $4526) at a threshold of $50,000 per QALY, $1173 (95% CI, −$2096 to $4442) at $100,000 per QALY, and $953 (95% CI, −$2604 to 4512) at $200,000 per QALY. Although point estimates suggested that cost savings outweighed the monetized health loss at these thresholds, the wide confidence intervals indicated substantial uncertainty around these estimates. The probability of cost-effectiveness decreased from approximately 80% to 71% as the decision thresholds increased from $20,000 to $200,000 per QALY, as demonstrated in the cost-effectiveness acceptability curve (Supplementary Fig. 4).

### Sensitivity analyses

In sensitivity analyses examining alternative approaches to rehabilitation ascertainment using grouped discharge-destination categories, the mean per-patient rehabilitation cost was $1564 ($3561) in the conservative oxygen group and $1403 ($3454) in the liberal oxygen group. This resulted in an incremental cost of $161 (95% CI, −$162 to $485). Incorporating this rehabilitation cost did not materially change the overall findings, and the point estimates for the total mean per-patient cost remained lower in the conservative oxygen group than in the liberal oxygen group (incremental cost −$1432, 95% CI, −$4744 to $1880).

Similarly, health outcome results were consistent between complete-case and multiple imputation analyses. After multiple imputation, the adjusted difference in QALYs was −0.003 (95% CI, −0.013 to 0.007), compared with −0.002 (95% CI, −0.013 to 0.008) in the complete-case analysis. The difference in favorable functional outcome was −1.6 percentage points (95% CI, −6.3 to 3.0) after multiple imputation and −1.5 percentage points (95% CI, −6.1 to 3.2) in the complete-case analysis.

### Sub-group analyses

Subgroup analyses by cardiac arrest cause (medical versus non-medical), by cardiac arrest location (OHCA versus IHCA), or by initial shockable rhythm showed similar treatment effect across subgroups for costs, favorable functional outcome, QALYs, or survival time (Supplementary Tables 4–6). For example, the incremental difference in mean per-patient cost between conservative and liberal oxygen therapy was −$5789 (95% CI, −$15,654 to $4076) among patients with non-medical arrest causes and −$830 (95% CI, −$4329 to $2669) among those with medical arrest causes. Corresponding differences in QALYs were −0.006 (95% CI, −0.034 to 0.021) and −0.002 (95% CI, −0.013 to 0.009), respectively.

## Discussion

We provide novel evidence on the cost-effectiveness of conservative compared with liberal oxygen therapy for adults with HIE after cardiac arrest. Conservative oxygen therapy was associated with modest cost savings and very small reductions in QALYs, life-years, and favorable functional outcomes. The magnitude of these differences was small (0.002 QALYs and 0.008 life-years, equivalent to less than 1 quality-adjusted life day and approximately 3 life days) and unlikely to be clinically meaningful. Consistent with this, most bootstrap replications were concentrated in the south-west quadrant and clustered close to the origin of the cost-effectiveness planes. Although the probability of cost-effectiveness remained above 70% across all evaluated decision thresholds up to $200,000 per QALY, these findings should be interpreted in the context of the very small differences in costs and outcomes, alongside the substantial uncertainty surrounding the estimates. Overall, these findings suggest that neither oxygen strategy offers a clear clinical or economic advantage.

We identified no published economic evaluations comparing conservative and liberal oxygen therapy in ventilated adults with HIE after cardiac arrest. However, similar findings were observed in the Oxy-PICU trial, which evaluated 1726 critically ill children across 15 pediatric ICUs in England and Scotland. Over one year of follow-up, the authors reported comparable QALYs and life-years between oxygen strategies, with conservative oxygen associated with modest cost savings.^32^ As in the present study, the economic differences between strategies were small. In adults, a comparable trial-based economic evaluation alongside the UK-ROX trial, which enrolled more than 16,500 invasively mechanically ventilated patients across 100 ICUs, has yet to be reported.^33^

This economic evaluation had several strengths, including the prespecified analysis plan, the large randomized trial population, and the use of patient-level data collected across multiple ICUs in Australia, New Zealand, and Ireland. The multinational design enhanced the generalizability of the findings across diverse critical care settings and reduced reliance on evidence from a single healthcare system. The analysis incorporated three complementary cost-effectiveness outcomes and was conducted and reported in accordance with the CHEERS guidelines.^34^ Together, these strengths increased confidence that the findings provided a reliable assessment of the economic implications of conservative and liberal oxygen therapy in this population.

Several limitations should be acknowledged in addition to those previously reported for the trial.^11^ Healthcare resource use was restricted to inpatient services, and follow-up was limited to 180 days. The exclusion of longer-term healthcare utilization after discharge could influence the cost-effectiveness results if post-discharge readmission patterns differ between oxygen strategies. Prior evidence suggests that nearly 20% of survivors are readmitted within 30 days of discharge, with an average of 1.85 readmissions per patient.^35^, ^36^ Additionally, incorporating broader health system costs, such as primary care services, and adopting a societal perspective, including informal care and productivity losses, may better capture the full economic burden of HIE after cardiac arrest. Furthermore, costs were estimated using Australian and New Zealand unit costs and healthcare delivery patterns, which may limit direct transferability to other jurisdictions. Nevertheless, the negligible intervention cost and minimal between-group differences in resource use suggest that the overall conclusions are likely applicable to healthcare systems with similar models of critical care. In low-resource settings where oxygen availability is constrained and oxygen supply represents a substantial incremental cost, differences in oxygen consumption between treatment strategies may have greater economic implications. Finally, although missing favorable functional outcome and EQ-5D-5L data were relatively limited (6–7%), they may have introduced additional uncertainty into the cost-effectiveness estimates; however, the results were consistent between complete-case and multiple imputation analyses.

## Conclusion

In adults with HIE after cardiac arrest, conservative oxygen therapy was associated with modest cost savings and minor, clinically negligible reductions in QALYs, life-years, and favorable functional outcomes. Due to the small magnitude of these differences and substantial uncertainty around the estimates, neither strategy demonstrates a clear clinical or economic advantage.

## Role of the funder/sponsor

The funder had no role in the design and conduct of the study; collection, management, analysis, and interpretation of the data; preparation, review, or approval of the manuscript; and decision to submit the manuscript for publication.

## Data sharing statement

The data underlying this article will be shared on reasonable request to the corresponding author.

## Code availability

The Stata codes used for the data analyses in this study will be made available upon reasonable request to the corresponding author.

## Use of AI

ChatGPT was used during manuscript preparation to assist with editing for conciseness and improving clarity of wording. No analyses, results, or interpretations were generated using AI.

## CRediT authorship contribution statement

**Zhomart Orman:** Writing – review & editing, Writing – original draft, Visualization, Validation, Methodology, Investigation, Formal analysis, Conceptualization. **Carol Hodgson:** Writing – review & editing, Supervision, Project administration, Funding acquisition, Data curation, Conceptualization. **Michael Bailey:** Writing – review & editing, Investigation, Funding acquisition, Formal analysis, Data curation. **Diane Mackle:** Writing – review & editing, Project administration, Funding acquisition, Data curation. **Anne M Mather:** Writing – review & editing, Project administration, Funding acquisition, Data curation. **Paul Young:** Writing – review & editing, Supervision, Project administration, Funding acquisition, Data curation, Conceptualization. **Alisa M. Higgins:** Writing – review & editing, Writing – original draft, Visualization, Validation, Supervision, Methodology, Investigation, Funding acquisition, Formal analysis, Data curation, Conceptualization.

## Funding

The cost-effectiveness analysis was conducted as part of the LOGICAL trial, which was funded by the Health Research Council of New Zealand (23/017), the Alpha Charitable Trust and the David & Cassie Anderson Medical Charitable Trust administered by the Perpetual Guardian Trust (all in New Zealand; G-202505-15015), the National Health and Medical Research Council (Australia; APP2001237), and the Irish Critical Care – Clinical Trials Network (Ireland; CTN-2021-010).

## Declaration of competing interest

The authors declare the following financial interests/personal relationships which may be considered as potential competing interests: Alisa M. Higgins is supported by an Investigator Grant (2008447) from the National Health and Medical Research Council of Australia, and is a Director of Empiric Heath. Carol Hodgson is supported by an Investigator Grant (2033103) from the National Health and Medical Research Council of Australia. If there are other authors, they declare that they have no known competing financial interests or personal relationships that could have appeared to influence the work reported in this paper.
