# Supplementary material for: Conservative versus liberal oxygen therapy in hypoxic ischemic encephalopathy following cardiac arrest: a trial-based cost-effectiveness analysis
Source: Resusc Plus. 2026 Jul 9;30:101403. doi: 10.1016/j.resplu.2026.101403 (PMC13393807; doi:10.1016/j.resplu.2026.101403)
Supplement: Supplementary Appendix [file mmc1.docx]

Supplementary Appendix

This Supplementary Appendix is provided by the authors to give readers additional information about the manuscript “Cost-effectiveness of conservative versus liberal oxygen therapy in hypoxic ischemic encephalopathy following cardiac arrest”.

**Table of Contents**

[Full list of LOGICAL Investigators 2](#_Toc225175856)

[Study Management Committee 2](#_Toc225175857)

[Statisticians 2](#_Toc225175858)

[Project Management Team 2](#_Toc225175859)

[Logical Investigators and Sites 2](#_Toc225175860)

[Funding 6](#_Toc225175861)

[Use of AI 6](#_Toc225175862)

[CHEERS 2022 Checklist 7](#_Toc225175863)

[Supplementary Methods 9](#_Toc225175864)

[Length of hospital stay measurement 9](#_Toc225175865)

[Handling missing data 9](#_Toc225175866)

[Inpatient rehabilitation measurement 10](#_Toc225175867)

[Rationale for exclusion of model-based economic evaluation 10](#_Toc225175868)

[Supplementary Results 11](#_Toc225175869)

[Supplementary Table 1 11](#_Toc225175870)

[Supplementary Table 2 15](#_Toc225175871)

[Supplementary Table 3 16](#_Toc225175872)

[Supplementary Table 4 17](#_Toc225175873)

[Supplementary Table 5. 18](#_Toc225175874)

[Supplementary Table 6. 19](#_Toc225175875)

[Supplementary Figures 20](#_Toc225175876)

# Full list of LOGICAL Investigators

## Study Management Committee

Carol L Hodgson*, Diane Mackle†, Anne M Mather, Richard Beasley, Rinaldo Bellomo (deceased), Stephen Bernard, Kathy Brickell, Adam M Deane, Glenn Eastwood, Simon Finfer, Seton Henderson, Alisa M Higgins, Jessica Kasza, Natalie J Linke, Edward Litton, Christine F McDonald, James Moore, Alistair D Nichol, Rachael Parke, Sandra Peake, Paul Secombe, Ian M Seppelt, Ary Serpa Neto, Tony Trapani, Andrew Udy, and Paul J Young†, for the LOGICAL management committee and the Australian and New Zealand Intensive Care Society Clinical Trials Group.

* Co-Chair of the Committee and Co-Chief Investigator

† Lead Project Manager

## Statisticians

Michael Bailey (unblinded statistician), Jessica Kasza (blinded statistician)

## Project Management Team

Medical Research Institute of New Zealand, Wellington, New Zealand: Niña Beehre, Bianca Crichton, Allie Eathorne, Anna Hunt, Diane Mackle, Cassie Lawrence, Mary La Pine, Shaanti Olatunji, Anne Turner. Australian and New Zealand Intensive Care Research Centre, Melbourne, Australia: Amanda Brown, Laura Johanson, Natalie J Linke, Anne M Mather, Liz Melgaard. The Irish Critical Care- Clinical Trials Network, University College, and Clinical Research Centre at St Vincent’s University Hospital, Dublin, Ireland: Kathy Brickell.

## Logical Investigators and Sites

***Australia***

Alfred Health: Laura Adams, Jasmin Board, Aidan Burrell, Phoebe McCracken, Emma Martin, David Pilcher, Andrew Udy, Meredith Young.

Angliss Hospital – Eastern Health: Graeme Duke, Kym Gellie, Niall Kennedy, Philippe Le Fevre, Dianne Parker, Nicole Robertson.

Austin Hospital: Rinaldo Bellomo, Glenn Eastwood, Ary Serpa Neto, Leah Peck, Helen Young.

Barwon Health, University Hospital Geelong: Allison Bone, Simone Fitzgerald, Ashley Garnett, Stacey Hawker, Michelle Horton, Stephanie Pearce, Paul Power, Jemma Trickey.

Bendigo Health: Catherine Boschert, Angus Carter, Cameron Knott, Julie Smith, Kim Woodger.

Box Hill Hospital – Eastern Health: Kate Cook, Graeme Duke, Kym Gellie, Niall Kennedy, Philippe Le Fevre, Nicole Robertson.

Caboolture Hospital: Julia Affleck, Andrew Murray, Mahesh Ramanan, Luke Reynolds, Emma Williams.

Cairns Hospital: Sussan Conway, Sarah Furness, Rajendra Goud, Catherine Tacon, Rahul Tambade.

Casey Hospital: Sandra Cork, Patrice Ganuelas, Umesh Kadam, Seema Radhakrishnan, Sheenam Soni.

Dubbo Base Hospital: Renae Bryant, Zoe Carr, Rachel Choit, Madeline Prestridge, Timothy Stanley, Maygen Weber.

Flinders Medical Centre: Shailesh Bihari, Julia Brown, JoAnne McIntyre, Shivesh Prakash.

Gold Coast University Hospital: Maree Houbert, James McCullough, Julie Pitman, Mandy Tallott, Jennie Walker.

Gosford Hospital: Christine Bailey, Shelley Donovan, Atul Gaur, Eloise Hair, Mary Keehan, Saara Mohamed, Elisha Turner.

Grampians Health: Dianne Brown, Andrea Burne, Khaled El-Khawas, Kate Flynn, Victoria Jennings, Angus Richardson, Courtney Rowe.

Ipswich Hospital: Neeraj Bhadange, Vijo Kuruvilla, Hannah McCabe, Wayne Stevens, Steven Tyler.

John Hunter Hospital: Sarah Dalton, Charley Keough, Rakshit Panwar, Amber-Louise Poulter, Krishna Sunkara, Torg Westerlund.

Lyell McEwin Hospital: Tim Beckingham, Natalie Soar, Melanie Wittwer.

Maroondah Hospital – Eastern Health: Graeme Duke, Kym Gellie, Niall Kennedy, Philippe Le Fevre, Peter Oziemski, Nicole Robertson.

Mater Misericordiae Ltd: Sadie Callahan, Claire Filet, Mackenzie Finnis, Gemma Hammond, Loki Johnk, Katherine Jongebloed, Nai An Lai, Adrian Langley, Isabel Anne Leditschke, Andras Nyikovics, Amar Pandey, Ravi Chockalingam Pillai, Peter Scott, Zander Tait.

Monash Medical Centre, Monash Health: David Antognini, Alana Brown, Alice Li, Chloe Peppin.

Nepean Hospital: Rebecca Gresham, Sheeja Joy, Julie Lowrey, Kristy Masters, Ian Seppelt.

Northern Hospital: David Crosbie, Angaj Ghosh, Simone Said.

Northern Beaches Hospital: Matthew Phillips, Lenae Terrill.

Orange Health Service: James Basha, Renae Bryant, Zoe Carr, Merryn Jones, Kerry Lenton, Fiona Shields, Maygen Weber.

Princess Alexandra Hospital: Megan Davis, Meg Harward, Josephine Mackay, Nicola McGuinness, Amanda McKee, Jason Meyer, James Walsham, Kyle White.

The Queen Elizabeth Hospital: Catherine Kurenda, Sandra Peake, Srilatha Vemparala, Patricia Williams, Nikki Yeo.

Royal Adelaide Hospital: Paola Arce-Arango, Imogen Asser, Nerissa Brown, Connor Christie, Sarah Doherty, Mahni Foster, Kathleen Glasby, Rhea Louis, Mark Plummer, Justine Rivett, Hope Smith.

Royal Darwin Hospital: Lewis Campbell, Alexandra Hinchcliff, Elizabeth Ross, Kirsty Smyth, Mary Tiras.

The Royal Melbourne Hospital: Deborah Barge, Alice Barrese, Kathleen Byrne, Adam Deane, Lewis Hackenberger, Barry Johnson, Christopher MacIsaac, Haustine Panganiban, Jeffrey Presneill, Thomas Rechnitzer, Elisa Zaccagnini-Hitchcock.

Royal North Shore Hospital: Christopher Andersen, Frances Bass, Lachlan Donaldson, Rosalind Elliot, Emily Fitzgerald, Oliver Flower, Tessa Garside, Naomi Hammond, Anne O’Connor, Wade Stedman, Elizabeth Yarad.

Royal Perth Hospital: Trisha Hardman, Sebastian Knudsen, Abhijit Laha, Sharon Waterson.

St George Hospital: Belinda Anderson, Chloe Edwards, Deborah Inskip, Kathryn Maloney, Chloe Moore, Shaun Parish, Manoj Saxena, Rebecca Sidoli, Sarah Valle, Frank Van Haren, Victoria Ying.

St Vincent’s Hospital, Sydney: Alexandra Campion, Amelia Hall, Sarah Henstridge, Amelia Liu, Stephen Morgan, Claire Reynolds.

St Vincent’s Hospital Melbourne: Alastair Brown, Jennifer Holmes, Melissa King, Viean Luk, Adelle Odering, Humphrey Walker.

Sunshine Coast University Hospital: Donna Appleton, Jane Brailsford, Peter Garrett, Moreblessing Kajevu, Samuel Marment, Lauren Murray, Tenelle Smith.

Toowoomba Hospital: Judy Smith, Adam Visser.

Victorian Heart Hospital: David Antognini, Alana Brown, Alice Li, Adrian Pakavakis, Roger Smith.

Westmead Hospital: David Bowen, Jing Kong, Cindy Liang, Catherine Linh, Angelica Monzon, Vineet Nayer.

Wollongong Hospital: Wenli Geng, Matthew MacPartlin.

Wyong Hospital: David Bowen, Leegan Cheung.

***Ireland***

St Vincent’s Hospital, Dublin: Kate Ainscough, Kathy Brickell, Alistair Nichol, Aideen Sharry, Biji Thomas.

***New Zealand***

Christchurch Hospital: Toby Bettridge, Tara Doyle, Jennifer Matthews, Jan Mehrtens, Stacey Morgan, Anna Morris, Kymbalee Van Der Heyden, Averil Whitworth.

Department of Critical Care Medicine, Auckland City Hospital: Lizzie Briggs, Yan Chen, Chris Hands, Archie Maclang, Colin McArthur, Rachael McConnochie, Kristian Misa, Hiromi Nakamuro, Caroline O’Connor, Catherine Simmonds, Lauren West.

Dunedin Hospital: Alisha DaSilva, Amie Eden, Dawn France, Robyn Hutchison, Christina Kumate, Pawel Twardowski.

Hawke's Bay Hospital: Llesley Chadwick, Ross Freebairn, Penelope Park, Christine Rolls.

Nelson Hospital: Alex Browne, Jette Koelle, Charlotte McNab.

North Shore Hospital: Hina Karim, Ywain Lawrey.

Rotorua Hospital: Ulrike Buehner, Siew-Bek Goh-Robinson, Arihia Waaka, Erin Williams.

Taranaki Base Hospital: Jonathan Albrett, Carolyn Jackson, Simon Kirkham, Cathy Vickers.

Tauranga Hospital: Caroline Abbott, Jonathan Chen, Jennifer Goodson, Amanda Miller.

Waikato Hospital: Amelia Butler, Annette Forrest, Fiona Gray, Pranesh Jogia, Jenny Karp, Robert Martynoga, Geoff McCracken, Jessica Newland, Suvarna Rathod, Mark Tan, Kara Trask, Dhongmei Zhao.

Wellington Regional Hospital: April Aguilar, Odessa David, Kirsha Delaney, Reece Latonio, Eden Lesona, Joy Marmol, Leanlove Navarra, Shan Qiu, Nina Roberts, Raulle Sol Cruz, Rhoze Sol Cruz, Paul Young.

Whangarei Hospital: Ralph Fuchs, Bridget Lambert, Kerstin Larsson, Ben Murrin.

# Funding

Funding for the LOGICAL trial was provided by the Health Research Council of New Zealand, the Alpha Charitable Trust, and the David & Cassie Anderson Medical Charitable Trust administered by the Perpetual Guardian Trust (all in New Zealand); the National Health and Medical Research Council (Australia); and the Irish Critical Care- Clinical Trials Network (Ireland).

# Use of AI

ChatGPT was used during manuscript preparation to assist with editing for conciseness and improving clarity of wording. No analyses, results, or interpretations were generated using AI.

# CHEERS 2022 Checklist

**Consolidated Health Economic Evaluation Reporting Standards (CHEERS) 2022 Checklist**

|  | **Item** | **Guidance for Reporting** | **Reported in section** |
| --- | --- | --- | --- |
| **TITLE** | | |  |
| Title | 1 | Identify the study as an economic evaluation and specify the interventions being compared. | Page 1 (Title page) |
| **ABSTRACT** | | |  |
| Abstract | 2 | Provide a structured summary that highlights context, key methods, results and alternative analyses. | Page 2 (Abstract) |
| **INTRODUCTION** | | |  |
| Background and objectives | 3 | Give the context for the study, the study question and its practical relevance for decision making in policy or practice. | Pages 4-5 (Introduction) |
| **METHODS** | | |  |
| Health economic analysis plan | 4 | Indicate whether a health economic analysis plan was developed and where available. | Page 5 (Methods) |
| Study population | 5 | Describe characteristics of the study population (such as age range, demographics, socioeconomic, or clinical characteristics). | Pages 5-6 (Methods – Trial design and participants) |
| Setting and location | 6 | Provide relevant contextual information that may influence findings. | Pages 5-6 (Methods – Trial design and participants) |
| Comparators | 7 | Describe the interventions or strategies being compared and why chosen. | Page 6 (Methods – Interventions) |
| Perspective | 8 | State the perspective(s) adopted by the study and why chosen. | Page 5 (Methods) |
| Time horizon | 9 | State the time horizon for the study and why appropriate. | Page 5 (Methods) |
| Discount rate | 10 | Report the discount rate(s) and reason chosen. | Page 5 (Methods) |
| Selection of outcomes | 11 | Describe what outcomes were used as the measure(s) of benefit(s) and harm(s). | Pages 6-7 (Methods – Health outcomes) |
| Measurement of outcomes | 12 | Describe how outcomes used to capture benefit(s) and harm(s) were measured. | Pages 6-7 (Methods – Health outcomes) |
| Valuation of outcomes | 13 | Describe the population and methods used to measure and value outcomes. | Pages 6-7 (Methods – Health outcomes) |
| Measurement and valuation of resources and costs | 14 | Describe how costs were valued. | Pages 7-8 (Methods – Cost estimation) |
| Currency, price date, and conversion | 15 | Report the dates of the estimated resource quantities and unit costs, plus the currency and year of conversion. | Pages 7-8 (Methods – Cost estimation) |
| Rationale and description of model | 16 | If modelling is used, describe in detail and why used. Report if the model is publicly available and where it can be accessed. | Supplementary Appendix - Rationale for exclusion of model-based economic evaluation |
| Analytics and assumptions | 17 | Describe any methods for analysing or statistically transforming data, any extrapolation methods, and approaches for validating any model used. | Pages 9-10 (Supplementary Appendix) |
| Characterizing heterogeneity | 18 | Describe any methods used for estimating how the results of the study vary for sub-groups. | Page 10 (Methods – Statistical analysis) |
| Characterizing distributional effects | 19 | Describe how impacts are distributed across different individuals or adjustments made to reflect priority populations. | Not undertaken |
| Characterizing uncertainty | 20 | Describe methods to characterize any sources of uncertainty in the analysis. | Pages 9-10 (Methods – Statistical analysis) |
| Approach to engagement with patients and others affected by the study | 21 | Describe any approaches to engage patients or service recipients, the general public, communities, or stakeholders (e.g., clinicians or payers) in the design of the study. | Pages 3-7 (Supplementary Appendix - Full list of LOGICAL Investigators) |
| **RESULTS** | | |  |
| Study parameters | 22 | Report all analytic inputs (e.g., values, ranges, references) including uncertainty or distributional assumptions. | n/a |
| Summary of main results | 23 | Report the mean values for the main categories of costs and outcomes of interest and summarise them in the most appropriate overall measure. | Pages 11-14 (Results) |
| Effect of uncertainty | 24 | Describe how uncertainty about analytic judgments, inputs, or projections affect findings. Report the effect of choice of discount rate and time horizon, if applicable. | Pages 12-14 (Results) |
| Effect of engagement with patients and others affected by the study | 25 | Report on any difference patient/service recipient, general public, community, or stakeholder involvement made to the approach or findings of the study | Not undertaken |
| **DISCUSSION** | | |  |
| Study findings, limitations, generalizability, and current knowledge | 26 | Report key findings, limitations, ethical or equity considerations not captured, and how these could impact patients, policy, or practice. | Pages 14-16 (Discussion) |
| **OTHER RELEVANT INFORMATION** | | | |
| Source of funding | 27 | Describe how the study was funded and any role of the funder in the identification, design, conduct, and reporting of the analysis | Pages 18 (Funding) |
| Conflicts of interest | 28 | Report authors conflicts of interest according to journal or International Committee of Medical Journal Editors requirements. | Page 18 (Conflicts of Interest) |

Husereau D, Drummond M, Augustovski F, de Bekker-Grob E, Briggs AH, Carswell C, Caulley L, Chaiyakunapruk N, Greenberg D, Loder E, Mauskopf J, Mullins CD, Petrou S, Pwu RF, Staniszewska S; CHEERS 2022 ISPOR Good Research Practices Task Force. Consolidated Health Economic Evaluation Reporting Standards 2022 (CHEERS 2022) Statement: Updated Reporting Guidance for Health Economic Evaluations. BMJ. 2022;376:e067975.

The checklist is Open Access distributed in accordance with the terms of the Creative Commons Attribution (CC BY 4.0) license, which permits others to distribute, remix, adapt and build upon this work, for commercial use, provided the original work is properly cited. See: [http://creativecommons.org/licenses/by/4.0/.](http://creativecommons.org/licenses/by/4.0/)

# Supplementary Methods

## Length of hospital stay measurement

Length of stay was defined as the total duration of inpatient care from randomization in the intensive care unit (ICU) during the index hospital admission until final discharge to a non-acute setting, death, or the end of follow-up. Transfers to another acute hospital were treated as a continuation of the index inpatient episode rather than as readmissions. Because length of stay at the receiving hospital was not observed, additional inpatient days following transfer were imputed using the mean length of stay among participants who were not transferred. For patients transferred to another hospital ICU, both ICU and subsequent medical ward days were added. For patients transferred directly to a medical ward, only additional ward days were added. For example, if a patient transferred to another ICU had an observed ICU stay of 2 days prior to transfer, and the mean ICU length of stay among non-transferred patients was 8 days, an additional 6 ICU days were imputed, followed by the mean medical ward length of stay. Although this assumption was not validated using external data, its impact on resource use estimates was small: mean ICU length of stay increased from 5.09 to 5.14 days and mean hospital length of stay from 6.68 to 6.92 days after imputation. Therefore, this assumption was unlikely to materially affect the cost-effectiveness results.

## Handling missing data

Missing data were summarized descriptively, except for the primary trial outcome (favorable functional outcome), which was reported in the main trial publication. Missingness of EQ-5D-5L utilities is described in this cost-effectiveness analysis and detailed in the Supplementary Appendix. Missing survival data at 180 days were less than 1% and were therefore not reported separately.

Missing favorable functional outcome and EQ-5D-5L utilities were imputed using multiple imputation by chained equations (m=20). EQ-5D-5L utilities were imputed using predictive mean matching, and favorable functional outcome using augmented logistic regression. The imputation model included treatment group, age, sex, ICU length of stay, and total costs. Quality-adjusted life years (QALYs) were calculated assuming baseline utility of 0 and linear interpolation to 180 days. Patients who died before 180 days were assigned a QALY of 0. Results from multiple imputation were compared with complete-case analyses.

## Inpatient rehabilitation measurement

Inpatient rehabilitation was identified using case report form data (yes/no) for participants in New Zealand, discharge destination indicating transfer to rehabilitation facilities in the APD for participants in Australia, and REDCap data for participants in Ireland. For patients discharged to another ICU or transferred to another hospital, rehabilitation status was not directly observed. Therefore, we assumed that their probability of subsequent discharge to inpatient rehabilitation was similar to that of patients not discharged to another ICU or hospital, and applied the corresponding rehabilitation proportions.

The impact of alternative approaches to ascertain inpatient rehabilitation data was examined in sensitivity analyses. Discharge destinations were grouped into clinically coherent categories reflecting post-acute care pathways: discharge to home; discharge to supported or community-based care (including nursing home, chronic care, mental health services, hospital-in-the-home, and other non-acute settings); and discharge involving ongoing acute hospital care (including patients remaining in hospital or transferred to another acute hospital or ICU). Rehabilitation rates observed among New Zealand participants were calculated within each grouped discharge-destination category and applied to participants in Australia and Ireland with corresponding discharge destinations to account for under-ascertainment of rehabilitation in APD/REDCap data.

## Rationale for exclusion of model-based economic evaluation

Although a model-based extrapolation was considered in the health economic analysis plan, it was not undertaken. The trial demonstrated no clinically meaningful or statistically significant differences in survival with a favorable functional outcome at 180 days, and there was no evidence to support differential long-term mortality or quality-of-life trajectories between groups. In the absence of robust data on post-180-day transition probabilities, long-term modelling would have required strong and unverifiable assumptions. Therefore, the within-trial analysis was considered the most appropriate and methodologically robust approach.

# Supplementary Results

## Supplementary Table 1. Unit costs applied to healthcare resource use for cost estimation (LOGICAL trial)

| Country | Cost item | Cost per unit, 2024 USD | Base year cost | Base year | Inflation rate (health/CPI) | PPP conversion factor (World Bank) | Sources |
| --- | --- | --- | --- | --- | --- | --- | --- |
| Australia | ICU stay | $4,315 per bed-day | AUD $5,760 per bed-day | 2023 | 4.9% | 1.4 | Independent Health and Aged Care Pricing Authority (2023). National Pricing Model 2023–24 <https://www.ihacpa.gov.au/sites/default/files/2023-03/national_pricing_model_technical_specifications_2032-24.pdf>  Australian Institute of Health and Welfare (2025) Health expenditure Australia 2023–24, AIHW, Australian Government, accessed 14 January 2026. <https://www.aihw.gov.au/reports/health-welfare-expenditure/health-expenditure-australia-2023-24/contents/overview/the-health-sector-relative-to-the-economy> |
| Australia | Medical ward (cardiac arrest) | $1,445 per bed-day | AUD $1,928 per bed-day | 2023 | 4.9% | 1.4 | Independent Health and Aged Care Pricing Authority (2023). National Hospital Cost Data Collection Public Sector 2022–23 [https://www.ihacpa.gov.au/resources/national-hospital-cost-data-collection-nhcdc-public-sectx`x`or-2022-23](https://www.ihacpa.gov.au/resources/national-hospital-cost-data-collection-nhcdc-public-sectx%60x%60or-2022-23)  Australian Institute of Health and Welfare (2025) Health expenditure Australia 2023–24, AIHW, Australian Government, accessed 14 January 2026. <https://www.aihw.gov.au/reports/health-welfare-expenditure/health-expenditure-australia-2023-24/contents/overview/the-health-sector-relative-to-the-economy> |
| Australia | Inpatient rehabilitation | $14,505 per case | AUD $19,359 per case | 2023 | 4.9% | 1.4 | Independent Health and Aged Care Pricing Authority (2023). National Hospital Cost Data Collection Public Sector 2022–23 [https://www.ihacpa.gov.au/resources/national-hospital-cost-data-collection-nhcdc-public-sectx`x`or-2022-23](https://www.ihacpa.gov.au/resources/national-hospital-cost-data-collection-nhcdc-public-sectx%60x%60or-2022-23)  Australian Institute of Health and Welfare (2025) Health expenditure Australia 2023–24, AIHW, Australian Government, accessed 14 January 2026. <https://www.aihw.gov.au/reports/health-welfare-expenditure/health-expenditure-australia-2023-24/contents/overview/the-health-sector-relative-to-the-economy> |
| New Zealand | ICU stay | $5,610 per bed-day | NZD $5,500 per bed-day | 2017 | 53% | 1.5 | PHARMAC Cost Resource Manual, Version 3. <https://pharmac.govt.nz/medicine-funding-and-supply/the-funding-process/policies-manuals-and-processes/economic-analysis/cost-resource-manual/>  Statistics New Zealand. Consumer Price Index Level 2 Subgroups for New Zealand. Hospital services. <https://infoshare.stats.govt.nz/ViewTable.aspx?pxID=9a5bbc07-50d7-44b2-aa1e-371c3a91a08c> |
| New Zealand | Medical ward (cardiac arrest) | $1,375 per bed-day | NZD $1,516 per bed-day | 2021 | 36% | 1.5 | WIESNZ20 (2020/21) // NZDRG70 codes: F76A-F76B <https://www.health.govt.nz/nz-health-statistics/data-references/weighted-inlier-equivalent-separations>  Statistics New Zealand. Consumer Price Index Level 2 Subgroups for New Zealand. Hospital services. <https://infoshare.stats.govt.nz/ViewTable.aspx?pxID=9a5bbc07-50d7-44b2-aa1e-371c3a91a08c> |
| New Zealand | Inpatient rehabilitation | $15,902 per case | AUD $19,359 per case | 2023 | 15% | 1.4 | Independent Health and Aged Care Pricing Authority (2023). National Hospital Cost Data Collection Public Sector 2022–23 <https://www.ihacpa.gov.au/resources/national-hospital-cost-data-collection-nhcdc-public-sector-2022-23>  Statistics New Zealand. Consumer Price Index Level 2 Subgroups for New Zealand. Hospital services. <https://infoshare.stats.govt.nz/ViewTable.aspx?pxID=9a5bbc07-50d7-44b2-aa1e-371c3a91a08c> |
| Ireland | ICU stay | $4,350 per bed-day | €2,659 per bed-day | 2008 | 30.9% | 0.8 | McLaughlin et al., 2009 <https://doi.org/10.1007/s00134-009-1622-1>  Central Statistics Office Ireland. Consumer Price Index. Hospital services. COICOP Division 06 Health. <https://www.cso.ie/en/statistics/prices/consumerpriceindex/> |
| Ireland | Medical ward (cardiac arrest) | $814 per bed-day | €626 per bed-day | 2022 | 4% | 0.8 | Healthcare Pricing Office. ABF 2022 Admitted Patient Price List. DRG codes: F76A F76B  Central Statistics Office Ireland. Consumer Price Index. Hospital services. COICOP Division 06 Health. <https://www.cso.ie/en/statistics/prices/consumerpriceindex/> |
| Ireland | Inpatient rehabilitation | $22,328 per case | €17,512 | 2023 | 2% | 0.8 | Healthcare Pricing Office. ABF 2023 Admitted Patient Price List. DRG codes: Z60A-Z60B  Central Statistics Office Ireland. Consumer Price Index. Hospital services. COICOP Division 06 Health. <https://www.cso.ie/en/statistics/prices/consumerpriceindex/> |

Abbreviations: AUD, Australian dollar; ABF, Activity-Based Funding; AIHW, Australian Institute of Health and Welfare; COICOP, Classification of Individual Consumption According to Purpose; CPI, Consumer Price Index; DRG, Diagnosis-Related Group; ICU, Intensive Care Unit; NZD, New Zealand dollar; PHARMAC, Pharmaceutical Management Agency (New Zealand); PPP, Purchasing Power Parity; WIESNZ, Weighted Inlier Equivalent Separation (New Zealand).

## Supplementary Table 2. Baseline characteristics of study participants with and without EQ-5D-5L data

| Characteristic | EQ-5D-5L available (n=1,692)* | EQ-5D-5L not available (n=129) |
| --- | --- | --- |
| Age, median (IQR) years | 63 (53-72) | 56 (46-65) |
| Female, n (%) | 477 (28.2) | 44 (34.1) |
| Country, n (%) |  |  |
| Australia | 1,181 (69.8) | 101 (78.3) |
| New Zealand | 508 (30.0) | 28 (21.7) |
| Ireland | 3 (0.2) | 0 (0) |
| Arrest cause, n (%) |  |  |
| Medical | 1,493 (88.2) | 102 (79.1) |
| Non-medical | 188 (11.1) | 21 (16.3) |
| Not recorded | 11 (0.7) | 6 (4.7) |
| Most prevalent diagnosis, n (%) |  |  |
| Ventricular tachycardia or fibrillation | 473 (28.0) | 36 (27.9) |
| Acute myocardial infarction | 286 (16.9) | 24 (18.6) |
| Pulseless electrical activity | 202 (11.9) | 7 (5.4) |
| Other | 731 (43.2) | 62 (48.1) |
| Cardiac arrest location, n/total n. (%) |  |  |
| Out-of-hospital | 1,256/1,684 (74.6) | 89/125 (71.2) |
| In-hospital | 428/1,684 (25.4) | 36/125 (28.8) |
| First monitored rhythm, n/total n. (%) |  |  |
| Shockable | 833/1,624 (51.3) | 67/108 (62.0) |
| Not shockable | 791/1,624 (48.7) | 41/108 (38.0) |

*EQ-5D-5L data were available for 1,692 patients, including 924 deceased patients who were assigned a utility score of zero.

Abbreviations: IQR, interquartile range.

## Supplementary Table 3. Baseline characteristics of study participants alive at follow-up with and without EQ-5D-5L data

| Characteristic | EQ-5D-5L available (n=768) | EQ-5D-5L not available (n=117) |
| --- | --- | --- |
| Age, median (IQR) years | 60 (51-70) | 56 (48-67) |
| Female, n (%) | 211 (27.5) | 40 (34.2) |
| Country, n (%) |  |  |
| Australia | 497 (64.7) | 89 (76.1) |
| New Zealand | 270 (35.2) | 28 (23.9) |
| Ireland | 1 (0.1) | 0 (0) |
| Arrest cause, n (%) |  |  |
| Medical | 694 (90.4) | 94 (80.3) |
| Non-medical | 66 (8.6) | 17 (14.6) |
| Not recorded | 8 (1.0) | 6 (5.1) |
| Most prevalent diagnosis, n (%) |  |  |
| Ventricular tachycardia or fibrillation | 293 (38.2) | 33 (28.2) |
| Acute myocardial infarction | 148 (19.3) | 23 (19.7) |
| Pulseless electrical activity | 55 (7.2) | 6 (5.1) |
| Other | 272 (35.3) | 55 (47.0) |
| Cardiac arrest location, n/total n. (%) |  |  |
| Out-of-hospital | 566/762 (74.3) | 79/113 (69.9) |
| In-hospital | 196/762 (25.7) | 34/113 (30.1) |
| First monitored rhythm, n/total n. (%) |  |  |
| Shockable | 507/723 (70.1) | 63/99 (63.6) |
| Not shockable | 216/723 (29.9) | 36/99 (36.4) |

Abbreviations: IQR, interquartile range.

## Supplementary Table 4. Summary of subgroup analyses by cause of cardiac arrest (medical vs non-medical)

| Outcome at 180 days | Conservative oxygen | | Liberal oxygen | | Incremental Difference  (95% CI) |
| --- | --- | --- | --- | --- | --- |
|  | n (%) | Mean (SD) | n (%) | Mean (SD) |  |
| Total mean per-patient cost |  |  |  |  |  |
| Non-medical | 101 (11.6) | $28,926 ($32,935) | 125 (13.2) | $34,715 ($42,908) | −$5,789 (−$15,654 to $4,076) |
| Medical | 772 (88.4) | $34,598 ($33,267) | 823 (86.8) | $35,428 ($37,905) | −$830 (−$4,329 to $2,669) |
| Favorable functional outcome (GOS-E 5-8) |  |  |  |  |  |
| Non-medical | 90 (11.0) | n/a | 113 (12.7) | n/a | −1.0 pp (−13.7 to 11.8) |
| Medical | 729 (89.0) | n/a | 777 (87.3) | n/a | −1.7 pp (−6.6 to 3.3) |
| QALYs |  |  |  |  |  |
| Non-medical | 88 (10.8) | 0.065 (0.109) | 111 (12.6) | 0.071 (0.110) | −0.006 (−0.034 to 0.021) |
| Medical | 725 (89.2) | 0.091 (0.108) | 768 (87.4) | 0.093 (0.109) | −0.002 (−0.013 to 0.009) |
| Survival time, days^†^ |  |  |  |  |  |
| Non-medical | 101 (11.6) | 76.0 (8.74) | 125 (13.2) | 85.9 (7.83) | −9.93 (−32.93 to 13.06) |
| Medical | 772 (88.4) | 92.8 (3.13) | 823 (86.8) | 95.0 (3.03) | −2.16 (−10.70 to 6.38) |

^*^P values are shown for interaction.

^†^Survival time was calculated as the restricted mean survival time (SE), truncated at 180 days.

Abbreviations: CI, confidence interval; GOS-E, Glasgow Outcome Scale Extended; pp, percentage points; QALYs, quality-adjusted life years; n/a, non-applicable; SE, standard error; SD, standard deviation.

## Supplementary Table 5. Summary of subgroup analyses by location of cardiac arrest (out-of-hospital vs in-hospital)

| Outcome at 180 days | Conservative oxygen | | Liberal oxygen | | Incremental Difference  (95% CI) |
| --- | --- | --- | --- | --- | --- |
|  | n (%) | Mean (SD) | n (%) | Mean (SD) |  |
| Total mean per-patient cost |  |  |  |  |  |
| IHCA | 221 (25.5) | $40,353 ($39,156) | 243 (25.8) | $43,633 ($48,435) | −$3,280 (−$11,261 to $4,700) |
| OHCA | 647 (74.5) | $31,674 ($30,816) | 698 (74.2) | $32,118 ($33,737) | −$445 (−$3,898 to $3,009) |
| Favorable functional outcome (GOS-E 5-8) |  |  |  |  |  |
| IHCA | 208 (25.5) | n/a | 221 (24.9) | n/a | −2.5 pp (−11.6 to 6.5) |
| OHCA | 607 (74.5) | n/a | 665 (75.1) | n/a | −1.2 pp (−6.6 to 4.2) |
| QALYs |  |  |  |  |  |
| IHCA | 208 (25.7) | 0.083 (0.105) | 220 (25.1) | 0.087 (0.105) | −0.004 (−0.024 to 0.015) |
| OHCA | 601 (74.3) | 0.094 (0.110) | 655 (74.9) | 0.096 (0.112) | −0.002 (−0.014 to 0.011) |
| Survival time, days^†^ |  |  |  |  |  |
| IHCA | 116 (25.8) | 92.3 (5.75) | 116 (24.5) | 98.5 (5.53) | −6.22 (−21.86 to 9.42) |
| OHCA | 333 (74.2) | 90.1 (3.45) | 357 (75.5) | 91.5 (3.31) | −1.39 (−10.75 to 7.98) |

^*^P values are shown for interaction.

^†^Survival time was calculated as the restricted mean survival time (SE), truncated at 180 days.

Abbreviations: CI, confidence interval; GOS-E, Glasgow Outcome Scale Extended; IHCA, in-hospital cardiac arrest; OHCA, out-of-hospital cardiac arrest; pp, percentage points; QALYs, quality-adjusted life years; n/a, non-applicable; SE, standard error; SD, standard deviation.

## Supplementary Table 6. Summary of subgroup analyses by shockable rhythm (shockable vs non-shockable)

| **Outcome at 180 days** | **Conservative oxygen** | | **Liberal oxygen** | | **Incremental Difference**  **(95% CI)** |
| --- | --- | --- | --- | --- | --- |
|  | **n (%)** | **Mean (SD)** | **n (%)** | **Mean (SD)** |  |
| Total mean per-patient cost |  |  |  |  |  |
| Non-sh. | 406 (49.0) | $30,667 ($32,305) | 426 (47.2) | $35,134 ($42,060) | −$4,467 (−$9,552 to $619) |
| Shock. | 423 (51.0) | $37,136 ($34,719) | 477 (52.8) | $34,895 ($33,802) | $2,241 (−$2,251 to $6,733) |
| Favorable functional outcome (GOS-E 5-8) |  |  |  |  |  |
| Non-sh. | 391 (49.7) | n/a | 402 (47.0) | n/a | −1.7 pp (−7.4 to 4.0) |
| Shock. | 395 (50.3) | n/a | 453 (53.0) | n/a | 0.3 pp (−6.4 to 7.1) |
| QALYs |  |  |  |  |  |
| Non-sh. | 389 (49.9) | 0.048 (0.092) | 402 (47.6) | 0.052 (0.091) | −0.004 (−0.017 to 0.009) |
| Shock. | 391 (50.1) | 0.132 (0.111) | 442 (52.4) | 0.128 (0.112) | 0.004 (−0.012 to 0.019) |
| Survival time, days^†^ |  |  |  |  |  |
| Non-sh. | 406 (49.0) | 57.3 (3.94) | 426 (47.2) | 63.2 (4.02) | −5.86 (−16.84 to 5.12) |
| Shock. | 423 (51.0) | 119.6 (3.45) | 477 (52.8) | 116.8 (3.82) | 2.87 (−8.00 to 13.75) |

^*^Risk differences for percentage points were estimated using binomial regression with identity link and may differ slightly from crude percentage differences, whereas incremental differences for means were estimated using linear regression.

^†^*P* values are shown for interaction (Cox proportional hazards models for survival time).

^‡^Survival time was calculated as the restricted mean survival time (SE), truncated at 180 days.

Abbreviations: CI, confidence interval; GOS-E, Glasgow Outcome Scale Extended; n/a, non-applicable; non-sh, non-shockable rhythm; pp, percentage points; QALYs, quality-adjusted life years; SE, standard error; SD, standard deviation; shock, shockable rhythm.

# Supplementary Figures

| **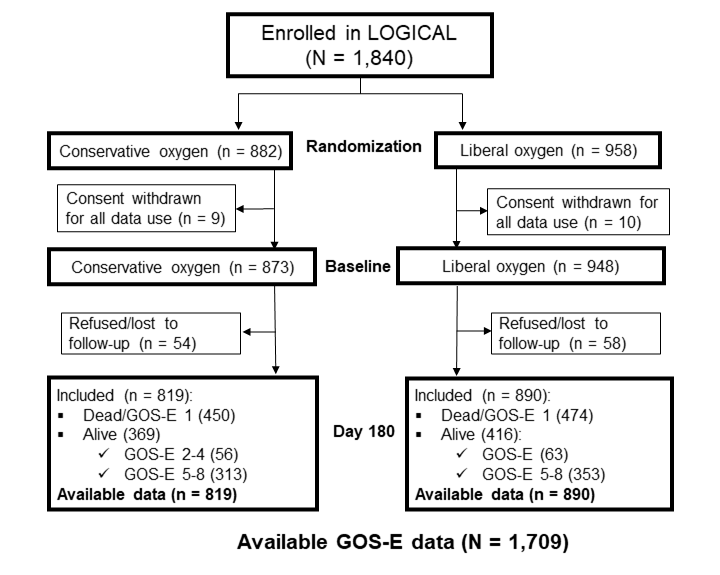** | |
| --- | --- |
| **Supplementary Figure 1. Flowchart of participants with Glasgow Outcome Scale Extended (GOS-E) data** | |
| **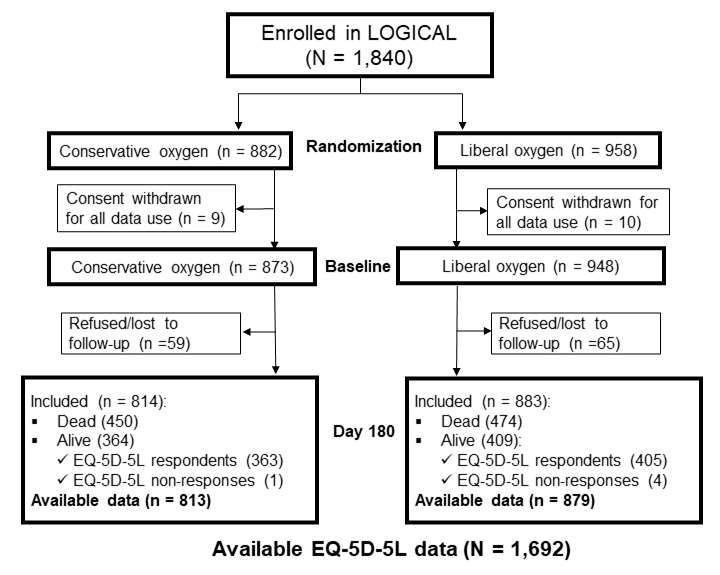** |  |
| **Supplementary Figure 2. Flowchart of participants with EQ-5D-5L data** |  |
| **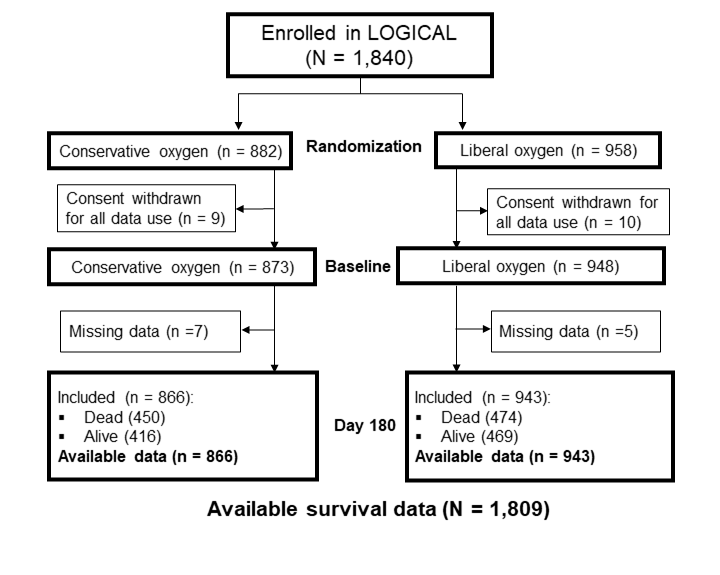** |  |
| **Supplementary figure 3. Flowchart of participants with survival data** |  |

| 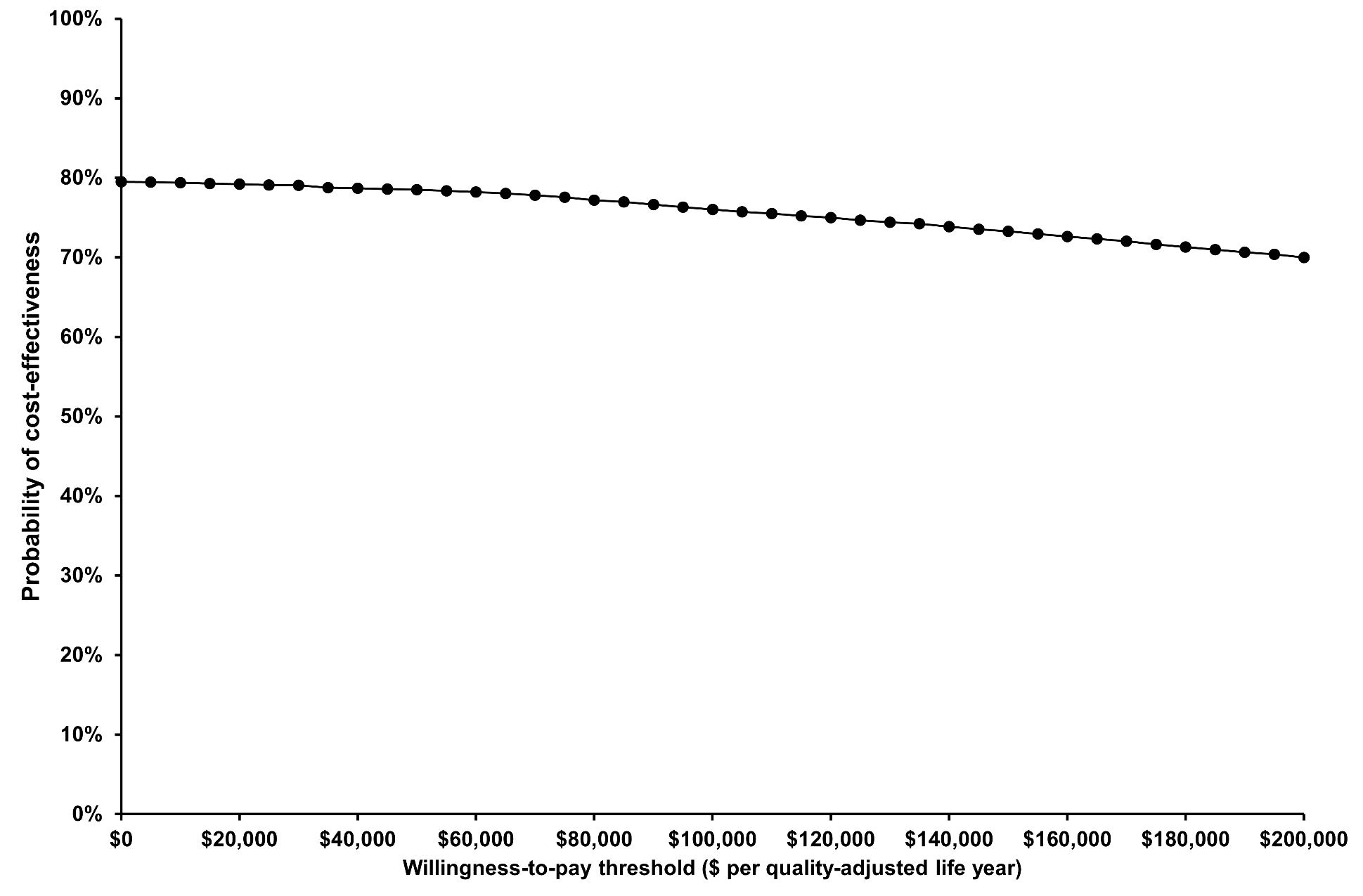 |
| --- |
| **Supplementary Figure 4. Cost-effectiveness acceptability curve** |
